# Teacher Versus Parent Informant Measurement Invariance of the Strengths and Difficulties Questionnaire

**DOI:** 10.1093/jpepsy/jsab062

**Published:** 2021-08-01

**Authors:** Aja Louise Murray, Lydia Gabriela Speyer, Hildigunnur Anna Hall, Sara Valdebenito, Claire Hughes

**Affiliations:** 1 Department of Psychology, University of Edinburgh; 2 Institute of Criminology, University of Cambridge; 3 Department of Psychology, Centre for Family Research, University of Cambridge

**Keywords:** ADHD, anxiety, attention, behavior problems, depression, hyperactivity, research design and methodology

## Abstract

**Background and Objectives:**

Obtaining a multi-informant perspective is important when assessing mental health issues in childhood and adolescence. Obtaining ratings from both parents and teachers also facilitates the evaluation of similarities and contrasts in the nature and severity of symptoms across home and school contexts. However, these informants may differ in their interpretations of observed behaviors, raising questions about the validity of comparing parents’ and teachers’ ratings.

**Methods:**

We evaluated the cross-informant measurement invariance of one of the most widely used measures of child and adolescent mental health: The Strengths and Difficulties Questionnaire (SDQ). Using data from the UK-population representative Millennium Cohort Study, we evaluated configural, metric, and scalar measurement invariance across parents and teachers when children were aged 7 (*N* = 10,221) and 11 (*N* = 10,543).

**Results:**

Scalar measurement invariance held at both ages. Parents reported higher levels of symptoms in all domains measured at both ages as well as higher prosociality.

**Conclusions:**

For a UK sample, valid comparisons of parent and teacher SDQ ratings at ages 7 and 11 appear to be possible, facilitating the evaluation of contextual differences in child mental health problems. Further, parents report more problem and prosocial behavior in their children than teachers attribute to them.

## Introduction

In some areas of mental health, such as attention-deficit/hyperactivity disorder (ADHD), clinical diagnosis depends upon demonstrating that symptoms occur across multiple contexts (American Psychiatric Association, 2013), necessitating the inclusion of multiple raters. In these and other areas obtaining a multi-informant perspective is also needed to document and explore potential differences in functioning across contexts and in interaction with different adults (De Los Reyes, 2013). This can help locate an issue to a particular context and thus guide the selection and targeting of interventions (Dirks et al., 2012). Indeed, it has been argued that encoding information about the context in which symptoms are expressed in diagnostic specifiers (e.g., subtypes defined by contexts of problems) may be a useful advance in future revisions of diagnostic criteria (Dirks et al., 2012; Murray et al., 2019b). Given the potential value of obtaining a multi-informant perspective on child and adolescent development, ratings are often collected from both teachers and parents in order to assess a young person’s functioning across the home and school context. However, questions remain regarding whether ratings from teachers and parents are psychometrically equivalent, permitting valid comparisons of symptom severity and relations with external factors (e.g., risk or protective factors) across settings/raters.

Disagreements between teachers’ and parents’ ratings of child psychopathology are common (e.g., Brown et al., 2006) and comparisons of mean symptom levels reported by these informants often reveal substantive systematic differences (Becker et al., 2004; Kennerley et al., 2018; Murray et al., 2018; Narad et al., 2015; Stone et al., 2010; Yeguez & Sibley, 2016). However, these comparisons rely on the implicit assumption that parents and teachers are providing equivalent ratings and are unlikely to be valid if parents and teachers have divergent interpretations of the meaning of the behaviors they observe. Such interpretational differences can lead, for example, to variations in the thresholds that need to be crossed for an informant to ‘count’ a behavior as a symptom.

A measurement invariance framework provides a useful approach to exploring potential differences in the way that parents and teachers understand and score mental health symptoms (Millsap, 2012). Measurement invariance across informants refers to the distribution of scores being independent of the informant providing them for a given underlying level of a construct (e.g., “emotional problems”). Though full measurement invariance (i.e., invariance of the entire distribution of scores for a given construct level) is difficult to assess, the confirmatory factor analysis (CFA) framework provides a tool for assessing a weaker version, namely, factorial invariance. CFA is increasingly being applied to shed light on informant, developmental, gender, pre- versus postintervention, country, and other important group differences in the context of child and adolescent mental health (Murray et al., 2017, 2019a; Stevanovic et al., 2015) and to establish whether it is valid to compare variances and means of constructs across these groups.

Although many widely used measures of child mental health include teacher and parent report versions, the issue of cross-informant measurement invariance has, to date, received relatively little attention. Relative to their widespread use, comparatively few studies having examined teacher- versus parent-informant invariance in major omnibus mental health measures such as the Child Behavior Checklist (CBCL), the Behavioral and Emotional Screening System (BASC), and the Strengths and Difficulties Questionnaire (SDQ), making it difficult to be sure that information obtained from these measures is comparable across these key informants (Konold et al., 2004; Rogge et al., 2018).

This study addresses cross-informant invariance in the SDQ (Goodman, 1997, 2001): one of the most widely used measures of child mental health globally. In pediatric contexts, it has been suggested that the SDQ can be used to increase the identification of mental health issues when used as a screen in primary care (Brown & Wissow, 2010) and to monitor outcomes for children whose medical history puts them at elevated risk of social, emotional, and behavioral problems (Bartal et al., 2020; Kyösti et al., 2019; Martinos et al., 2018). The instrument has five subscales measuring conduct problems, ADHD symptoms, emotional problems, peer problems, and prosociality. The SDQ was designed for 3–16 year olds, has been translated into approximately 80 languages, and is brief and easy to administer, making it a measure of choice for large scale studies as well as clinical and educational intervention studies globally (Sosu & Schmidt, 2017).

Although the psychometric properties of the SDQ scores have been extensively evaluated (Kersten et al., 2016; Stone et al., 2010), its measurement invariance across informants has received only limited exploration. Using a subset of 15 items from the SDQ, Deutz et al. (2018) demonstrated cross-informant measurement invariance up to the scalar level in a model of children’s behavioral and emotional dysregulation in a sample of youth covering early childhood to adolescence (mean age 6–13). However, they did not test measurement invariance in the 5D model that corresponds to the design and primary use of the SDQ. Rogge et al. (2018) examined the cross-informant measurement invariance of the 5D model of the SDQ and showed that scalar measurement invariance held across teachers and parents; however, they focused solely on the earlier years of childhood (ages 3–6). No study has yet—to the best of our knowledge—examined teacher–parent measurement invariance in the 5D SDQ model in older children and adolescents. This is important because previous research suggests that the magnitude and direction of informant differences may depend on age (Murray et al., 2018), and an analysis of measurement invariance across not only age (as has been previously established for the SDQ in the current sample; Murray et al., 2021a), but also informant is essential to illuminate these differences. Without such an analysis, it is not clear whether this simply reflects cross-informant measurement differences across age. In this study, we therefore examine cross-informant measurement invariance of the SDQ in both childhood (age 7) and early adolescence (age 11) in a large UK population-representative study.

## Materials and Methods

### Participants and Procedure

Participants were from the UK-based Millennium Cohort Study (MCS; Connelly & Platt, 2014). MCS follows children born at the beginning of the 21st century from age 9 months, with data currently available up to age 17. A stratified cluster sampling procedure was used to select participants living within all four nations of the UK. The sample is drawn from the population of children born between the dates of September 1, 2000 and August 31, 2001 for England and Wales and between the dates of November 24, 2000 and January 11, 2002 for Scotland and Northern Ireland. A key goal of the MCS was to ensure that the sample would allow for analyses of the effect of disadvantage and ethnicity, therefore, MCS oversampled socially disadvantaged families and families from areas of high ethnic minority concentration Sampling was then clustered by electoral wards and for selected wards children were identified based on the Child Benefit register (a universal provision at the time). Sensitive cases were excluded, for example, where children had died or been taken into local authority care, or where there was an investigation into benefit fraud. Further, families were excluded if they had already taken part in the Families and Children (FACS) survey. A small number of eligible children who were not initially identified via Child Benefit records were added to the sample based on identification by Health Visitors. Further eligible children who had not been identified by either method but who were later identified were added at the second wave. Demographic characteristics of the sample used in this study are provided in Table I.

**Table I.** Sample Demographic Descriptive Statistics

Variable	Category	%	*N*
Sex	Female	49.97	5,809
	Male	50.03	5,817
Child ethnicity	White	83.17	9,320
	Other ethnicity	16.83	1,886
Maternal academic qualification	Higher degree	3.98	446
	First degree	15.59	1,750
	Diplomas in higher education	9.52	1,066
	A/AS/S levels	10.01	1,121
	O level/GCSE Grades A–C	32.67	3,657
	GCSE Grades A–C	9.84	1,102
	Other academic qualification	2.74	307
	None of these qualifications	15.64	1,751
Deprivation	Most deprived decile	13.80	1,482
	10% to <20%	12.23	1,313
	20% to <30%	11.19	1,202
	30% to <40%	9.87	1,060
	40% to <50%	9.53	1,023
	50% to <60%	8.78	943
	60% to <70%	7.83	841
	70% to <80%	7.96	855
	80% to <90%	9.13	980
	Least deprived decile	9.68	1,039
		Mean	SD
Age	Age 7 Sweep	7.23	0.25
	Age 11 Sweep	10.67	0.48

*Note.* These are unweighted and based on the sample of participants with SDQ data at age 7 and/or 11 who took part in the MCS up until age 14, which was the latest measurement wave available at time of analysis.

Given the complex survey design involving unequal selection probabilities, design weights are provided to correct the sample estimates to representativeness, and stratification and clustering variables are provided to adjust parameter variance estimates (see e.g., Murray et al., 2021b). Design weights are also incorporated into the attrition weights used to correct for nonrandom drop-out over the course of the study and were derived using the predicted values from logistic regression models predicting drop-out from available data. They are used to effectively up-weight those with a low participation probability to counteract the bias introduced by non-random selection (see e.g., Seaman & White, 2013). Full information on MCS, including questionnaires and technical reports can be found at: https://ukdataservice.ac.uk.

This used data from waves 4 (*N* = 10,221) and 5 (*N* = 10,543), when the participants were aged 7 and 11 respectively. In the case of parent reports, in the vast majority of cases the child’s biological mother served as the “main respondent” to the parent-reported SDQ used in the current analysis. The SDQ was included as part of a self-completion module in the main respondent interviews. Teachers provided data on each child via a postal self-completion questionnaire (or in sweep 5 by telephone follow-up if no response was achieved by post). The technical documentation for MCS reports teacher survey response rates at age 7 and 11 of 70% and 77% respectively, with most nonresponse due to a lack of response from teachers even after two reminders; however, it should be noted that the latter wave only included teacher surveys for England and Wales due to resource constraints.

### Measures

The *Strengths and Difficulties Questionnaire (SDQ)* comprises five subscales measuring conduct problems, hyperactivity/inattention, emotional problems, prosociality, and peer problems. Each subscale includes five items with responses provided on a 3-point scale from (*not true*) to (*certainly true*). Respondents are also offered a “can’t say” and “not applicable” option. The psychometric properties of the SDQ have been well-studied and are reviewed by Kersten et al. (2016). In terms of factor structure, a majority of studies have supported the convergent and structural validity of a 5D model with dimensions corresponding to the above-described subscales. In this study, identical questions were asked of parents and teachers and at ages 7 and 11.

### Statistical Procedure

We used a confirmatory factor analysis (CFA) approach to evaluate cross-informant measurement invariance in the SDQ. Given the design of the SDQ and previous evidence that a 5D oblique factor model provides a good factor model for its items (Kersten et al., 2016), we adopted this as our factor structure in this study. In the configural model, latent factors were specified for emotional problems, conduct problems, hyperactivity/inattention, prosociality and peer problems for each informant. Weighted least squares means and variances (WLSMVs) estimation with theta parameterization was used to account for the ordered categorical response scale of items. For scaling and identification, the factor means and variances of each parent-reported construct were fixed to 0 and 1, respectively. In addition, one loading for each construct and one threshold for each item was fixed equal across informants. An additional threshold was fixed equal across informants for the items with loadings were fixed equal across informants (the “reference indicator”). Finally, residual factor variances were fixed equal to 1 for all items.

If this initial model fit reasonably well, configural measurement invariance was judged to hold. Metric measurement invariance was then tested by evaluating whether the addition of cross-informant equality constraints on factor loadings led to a substantial deterioration in fit. Assessing whether there is a substantial deterioration in fit is not straightforward. In principle, chi-square difference testing can be used but this technique has been shown to be sensitive to even trivial mis-specifications in large samples (such as the present sample; Yuan & Chan, 2016). The main proposed alternative is to compare fits based on statistics such as CFI, RMSEA, and SRMR. However, previous simulation studies have provided somewhat conflicting advice on appropriate thresholds for changes in these statistics, reflecting the fact that their sensitivity to noninvariance seems to depend on factors such as sample size, number of groups, item response scales, number of latent factors, and location and nature of noninvariance (Chen, 2007; Rutkowski & Svetina, 2017; Svetina & Rutkowski, 2017). Although no previous simulation study has examined the particular combination of factors we have in the present analyses, they can be used to guide the selection of thresholds that will be sensitive to important mis-specifications but avoid concluding that measurement invariance violations are trivially small and unlikely to be causing considerable bias in comparisons of reports across informants. We, therefore, adopted the widely used criteria suggested by Chen (2007) that are based on a comprehensive simulation study. Specifically, metric measurement invariance was judged to hold if CFI decreased by no more than 0.010; RMSEA increased by no more than 0.015, and SRMR increased by no more than 0.030 with the addition of these constraints (Chen, 2007). If metric measurement invariance did not hold, modification indices and expected parameter changes were used to guide the iterative release of cross-informant equality constraints to attempt to achieve a partially invariant metric model with at least two invariant items (Pokropek et al., 2019; Van de Schoot et al., 2012).

If a (partially) metric invariant model could be found, scalar measurement invariance was then tested by fixing all remaining free item thresholds to equality across informants, excepting any items that failed to show metric invariance. Scalar measurement invariance was judged to hold if CFI decreased by no more than 0.010, if RMSEA increased by no more than 0.015, and SRMR increased by no more than 0.010 with the addition of these constraints (Chen, 2007). If scalar measurement invariance did not hold, modification indices and expected parameter changes were consulted to guide the iterative release of constraints to attempt to achieve a partially invariant scalar model. Respondent-level missing data was dealt with using attrition weighting. This approach provides unbiased parameter estimates under “missing at random” (MAR) mechanisms (Rubin, 1976) that is, when missingness is random conditional on the modeled variables.

Based on the final models at age 7 and 11, omega internal consistency values were calculated (McDonald, 1999). Omega provides a conceptually similar measure of internal consistency to Cronbach’s alpha; however, it does not assume that all items have equal loadings within a factor.

## Results

### Descriptive Statistics

Descriptive statistics for the SDQ items are provided in Supplementary Tables S1 and S2. These show the weighted number of responses in each response category. In general, these indicate that the sample showed relatively low levels of symptoms, consistent with its normative nature.

### Cross-Informant Measurement Invariance at Age 7

Fits for all models are provided in Table II. The configural model for parent and teacher SDQ scores at age 7 fit reasonably well (CFI = 0.950, RMSEA = 0.018, and SRMR = 0.063). Imposing metric measurement invariance constraints resulted in a significant chi-square difference test and a slight deterioration in fit when taking CFI, RMSEA, and SRMR together (CFI = 0.950, RMSEA = 0.019, and SRMR = 0.067). The CFI, RMSEA, and SRMR deterioration was; however, not large enough to suggest a lack of measurement invariance according to our predefined criteria. Imposing scalar measurement invariance constraints was also associated with a significant chi-square test and led to a deterioration in fit in the other indices as well; however, the deterioration was within our predefined limits (CFI = 0.947, RMSEA = 0.019, and SRMR = 0.067). We could, therefore, conclude that scalar measurement invariance held. Based on this model, omega internal consistency values for the subscales were: .83 for emotional symptoms, .81 for conduct problems, .88 for hyperactivity/inattention. .86 for prosociality, and .82 for peer problems, all suggestive of high reliability.

**Table II.** Model Fits For the Cross-Informant Measurement Invariance Models

	Model fit			Fit difference						Link to full model output
Model	CFI	RMSEA	SRMR	$\Delta \chi^{2}$	*df*	*p*	ΔCFI	Δ RMSEA	Δ SRMR	
Age 7										
Configural	0.950	0.018	0.063	—	—	—	—	—	—	https://osf.io/x4vzn/
Metric	0.950	0.019	0.067	501.226	20	<.001	0.000	−0.001	−0.004	https://osf.io/u9mrx/
Scalar	0.947	0.019	0.067	1,409.400	20	<.001	0.003	0.000	0.000	https://osf.io/btq6v/
Age 11										
Configural	0.949	0.018	0.061	—	—	—	—	—	—	https://osf.io/we6qg/
Metric	0.949	0.017	0.064	285.378	20	<.001	0.000	0.001	−0.003	https://osf.io/s4rva/
Scalar	0.946	0.018	0.064	852.497	20	<.001	0.003	−0.001	0.000	https://osf.io/va53c/

*Note.* Metric measurement invariance is judged to hold if CFI decreases by no more than 0.010; RMSEA increases by no more than 0.015, and SRMR increases by no more than 0.030 with the addition of loading equality constraints and scalar measurement invariance is judged to hold if CFI decreases by no more than 0.010, if RMSEA increases by no more than 0.015, and SRMR increases by no more than 0.010 with the addition of threshold equality constraints.

Full model results can be found at: https://osf.io/3jfmv/files//. In the final model, teachers reported lower levels of emotional symptoms (standardized mean difference [SMD] = −0.24; *p* < .001, lower levels of conduct problems [SMD] = −0.70, *p* < .001), lower levels of hyperactivity/inattention (SMD = −0.29, *p* < .001), and lower levels of prosociality (SMD = −0.19, *p* < .001) than parents but no difference on peer problems (SMD = −0.03, *p* = .476). These SMDs are based on a standardization in which the variances of both the teacher and parent latent factors are set to 1. The correlations between parent and teacher ratings were *r* = .38 (*p* < .001) for emotional problems, *r =* .55 (*p* < .001) for conduct problems, *r* = .57 (*p* < .001) for hyperactivity/impulsivity, *r* = .35 (*p* < .001) for prosociality, and *r* = .56 (*p* < .001) for peer problems.

### Cross-Informant Measurement Invariance at Age 11

The configural model for parent and teacher-reported SDQ scores at age 11 also fit reasonably well (CFI = .949, RMSEA = 0.018, and SRMR = .061). The addition of metric measurement invariance constraints led to no change in CFI, a slight improvement in RMSEA, and a slight worsening of SRMR (CFI = 0.949, RMSEA = 0.017, and SRMR = 0.067), the latter being below Chen’s (2007) recommended threshold. This suggested that metric measurement invariance held. Adding scalar measurement invariance constraints to the model led to a slight deterioration in fit (CFI = 0.946, RMSEA = 0.018, and SRMR = 0.064); however, this remained within the bounds of Chen’s (2007) recommended criteria. Scalar measurement invariance was, therefore, also judged to hold. Omega internal consistency values based on this model were: .86 for emotional problems, .85 for conduct problems, .86 for hyperactivity/inattention, .86 for prosociality, and .84 for peer problems, again all suggestive of high reliability.

Full model results can be found at https://osf.io/3jfmv/files/In this model, teachers reported lower levels of emotional problems (SMD = −0.38; *p =* .001), conduct problems (SMD = −0.76; *p* < .001), hyperactivity/inattention (SMD = −0.43; *p* < .001), prosociality (SMD = −0.27, *p* < .001) and peer problems (SMD = −0.18; *p* < .001). The correlations between teacher and parent reports were: *r =* .47 (*p* < .001) for emotional problems; *r* = .63 (*p* < .001) for conduct problems; *r* = .62 (*p* < .001) for hyperactivity/impulsivity; *r* = .40 (*p* < .001) for prosociality; and *r =* .69 (*p* < .001) for peer problems.

## Discussion

Obtaining psychometrically comparable parent and teacher scores on child and adolescent psychopathology is necessary to enable cross-context comparisons of symptoms to guide interventions focusing on where particular problems manifest. In some areas they are also necessary to demonstrate pervasiveness for the purpose of obtaining a clinical diagnosis. In this study, we evaluated the measurement invariance of one of the most widely used measures of child and adolescent psychopathology globally: the SDQ across parents and teachers when rating young people at ages 7 and 11. We found that configural, metric, and scalar measurement invariance held across these informants at both ages. This suggests that the SDQ can be used to validly compare mean parent and teacher ratings of young people’s emotional problems, conduct problems, hyperactivity/inattention, prosociality, and peer problems. When compared with parents, teachers in the current sample tended to report lower levels of all constructs than parents (both problems such as emotional and conduct problems and a strength: prosociality) at both ages, the one exception being a lack of a difference in informant reports of peer problems at age 7. Despite these mean differences parent–teacher correlations were in the moderate to strong range, indicating that parents and teacher ratings tend to be in general agreement about where children and adolescents stand on their symptoms relative to others.

Taken together, our results support the use of the SDQ as a means of obtaining multi-informant data on child and adolescent mental health. The fact that configural and metric measurement invariance held suggests that the parent and teacher versions of the SDQ capture the same constructs, consistent with the idea that parents and teachers appear to share a common view of the meaning of emotional problems, conduct problems, hyperactivity/inattention, prosociality, and peer problems as they are operationalized in the SDQ. This measurement invariance is arguably a testament to the highly rigorous development process that underpins the SDQ (Goodman, 1997, 2001) as well as the measure’s minimal use of technical language that enables informants of varying backgrounds and levels of expertise to apply items to child behavior. Together with previous demonstrations that the SDQ shows longitudinal and gender measurement invariance across the age range of 5–14 in the same sample (Murray et al., 2021a) our findings of cross-informant measurement invariance suggests that the SDQ provides scores that can be validly compared across development, gender, and informants. Its comparability across these factors, its brevity (25 items), and ease of administration, makes the SDQ an excellent practical choice for large-scale research studies where full clinical assessments may not be feasible. These same properties also suggests the SDQ is likely to be an optimal choice of measure for use in clinical contexts such as in screening for mental health issues in pediatric care (Brown & Wissow, 2010). In these contexts, the SDQ provides a quick and feasible method of obtaining ratings from both parents and teachers to provide an indication of whether problems may be evident in the home and/or school context and suggest whether referral for fuller assessment may be beneficial. For the same reasons, it is likely to offer an optimal choice of omnibus assessment to monitor mental health sequelae of physical illness in pediatric populations.

Our findings of scalar measurement invariance is consistent with the limited previous research on the cross-informant measurement invariance of the SDQ in childhood (Rogge et al., 2018) and extend this conclusion to early adolescence. Demonstrating cross-informant measurement invariance represents an important advantage of the SDQ as a multi-informant instrument. This means that within the context of a latent measurement model, parent- and teacher-reported means can be validly compared (Liu et al., 2017). Such comparisons may be informative for illuminating the contexts in which particular problems are more likely to be detected. For example, these analyses suggested that for both 7- and 11-year olds, parents rated conduct and hyperactivity/inattention problems as higher than teachers. This is consistent with clinical observations that significant problems in the home are not always accompanied by problems in the school context (Rettew et al., 2011). One possible explanation for this contrast is that disruptive behaviors may be more evident within the relatively unstructured environment of the home (Murray et al., 2018).

Improved knowledge of normative parent–teacher differences are also helpful as there is some debate as to how best to combine the scores from teachers and parents in order to identify whether a young person is displaying problematic levels of symptoms in a particular domain (Dirks et al., 2012; Kennerley et al., 2018; Yeguez & Sibley, 2016). By showing the differences that would be, on average, expected to be observed between these informants, these findings provide a useful reference to aid the interpretations of discrepancies between parent and teacher ratings. For example, a child showing a pattern of more behavior problems at school might be considered to be showing an atypical pattern, indicating that assessments to identify, and interventions to address potential problems in the school context would be a higher priority than interventions focused on family functioning or parenting. Further research examining individual level patterns of discrepancy and their predictors/outcomes will be helpful to provide further illumination on this issue, with measurement invariance providing a critical foundation for these types of investigations (de Haan et al., 2018; Murray et al., 2019b).

The correlations between parent and teacher reports were in the moderate to strong range, suggesting that parents and teachers are generally in agreement regarding the constructs measured by the SDQ. As expected, correlations were stronger for the more overt symptoms of conduct problems and hyperactivity/inattention than for emotional problems (Rogge et al., 2018). The associations observed were in fact somewhat larger than the cross-informant associations often reported which are often only around *r = .*30 (Achenbach, 2006; Kennerley et al., 2018). Although this partly reflects the straightforward and nontechnical construction of the SDQ items, it will also reflect our use of a latent measurement model, which allows the associations between informant scores to be corrected for measurement error. However, the fact that the correlations were still far from unity even after correction further underlines the fact that reliance on multiple informants remains important to capture context-specific issues (De Los Reyes, 2013).

### Limitations and Future Directions

Some limitations of this study deserve note. First, our assessment of cross-informant measurement invariance was limited to teachers and parents. Self- and peer- reported data may be especially relevant for older age groups and useful for capturing information in unsupervised contexts (Clemans et al., 2014). Analyses of other inventories suggest potential violations of measurement invariance across self- and parent-reports (Olino et al., 2018), underlining the importance of evaluating it across these informants. Similarly, teacher data were available only at ages 7 and 11; therefore, we could not evaluate whether the cross-informant measurement invariance of the SDQ holds across the full range of ages for which the SDQ is designed to be used (i.e., ages 3–16). Second, although measurement invariance is consistent with items functioning the same across groups, it does not guarantee this (Widaman et al., 1992). Demonstrating measurement invariance is thus only one aspect of ensuring the comparability of informant scores. Cognitive interviews may also be helpful in illuminating potential differences in interpretation across informants (Collins, 2003). Third, the SDQ is available in numerous languages and used all over the world, but our analyses do not speak to the question of whether informant measurement invariance would hold in other countries. For example, in the UK context, dialogue between parents and teachers may be stronger than in settings where parents themselves have little formal schooling. This may promote a higher level of shared understanding and agreement between UK informants than may be achieved in some other countries. Fourth, we focused on cross-informant invariance using a single omnibus measure of mental health. Further research to establish whether these results generalize to other popular omnibus measures of child and adolescent mental health of differing designs will be important. When compared with some other popular omnibus measures, the SDQ can be considered a relatively brief and general measure. Instruments that provide more in-depth characterizations of mental health phenotypes and their subdimensions (e.g., better differentiation of inattention and hyperactivity, of anxiety and depression, or of aggressive and nonaggressive conduct problems) may be more liable to reveal measurement invariance violations. Finally, we examined parent-teacher measurement invariance with respect to continuously measured scores on the SDQ; however, an important future direction would be to examine concordance and differences between informants in whether youth cross clinical thresholds on the SDQ subscales. There is no universal consensus on optimal clinical cut-points for the SDQ subscales. This is because optimal cut-points are typically context dependent and will depend on whether in a given setting there are greater concerns regarding the avoidance of false positives versus negatives (e.g., due to varying base rates of mental health disorders). As such, these investigations should be sensitive to the specific contexts in which the SDQ may be being used.

## Conclusion

The SDQ scores show cross-informant configural, metric and scalar measurement invariance across parents and teachers when assessing 7- and 11-year olds, suggesting that the SDQ can be used to validly compare parent- and teacher- reported emotional problems, conduct problems, hyperactivity/inattention, prosociality, and peer problems in these age groups.

## Supplementary Data

Supplementary data can be found at: https://academic.oup.com/jpepsy.

*Conflicts of interest*: None declared.

## Supplementary Material

jsab062_Supplementary_Data
