# Supplementary material for: Teacher Versus Parent Informant Measurement Invariance of the Strengths and Difficulties Questionnaire
Source: J Pediatr Psychol. 2021 Aug 1;46(10):1249–57. doi: 10.1093/jpepsy/jsab062 (PMC8561254; doi:10.1093/jpepsy/jsab062)
Supplement: jsab062_Supplementary_Data [file jsab062_Supplementary_Data.docx]

**Table S1: Descriptive statistics (weighted Ns by response category) for SDQ items at age 7**

| **Item** | **Teacher reports** | | | **Parent reports** | | |
| --- | --- | --- | --- | --- | --- | --- |
| **Paraphrase of item content** | **Not**  **true** | **Somewhat true** | **Certainly true** | **Not**  **true** | **Somewhat true** | **Certainly true** |
| complains of headaches, stomach aches, sickness | 5498.288 | 982.868 | 241.724 | 7861.759 | 1732.767 | 434.084 |
| often seems worried | 4189.85 | 2124.092 | 402.129 | 7578.703 | 1941.497 | 422.606 |
| often unhappy | 5392.975 | 1058.346 | 279.315 | 8631.055 | 1133.039 | 279.234 |
| nervous or clingy in new situations | 4503.290 | 1800.662 | 424.992 | 5525.380 | 3436.434 | 1054.034 |
| many fears, easily scared | 5385.911 | 1107.618 | 196.569 | 7014.721 | 2396.251 | 554.890 |
| often has temper tantrums | 5965.182 | 529.265 | 237.881 | 5366.637 | 3237.856 | 1430.304 |
| generally obedient | 4955.018 | 1517.922 | 251.968 | 5874.119 | 3808.677 | 385.280 |
| fights with or bullies other children | 5779.896 | 758.125 | 189.587 | 9200.592 | 675.583 | 145.462 |
| steals from home, school or elsewhere | 6455.765 | 160.008 | 49.843 | 9723.354 | 225.436 | 115.536 |
| often lies or cheat | 5799.548 | 749.094 | 163.759 | 7559.224 | 2090.323 | 313.757 |
| restless, overactive, cannot stay still long | 4808.190 | 1301.323 | 623.844 | 5683.706 | 2614.409 | 1739.423 |
| constantly fidgeting | 4965.058 | 1182.750 | 582.838 | 5974.733 | 2795.669 | 1200.394 |
| easily distracted | 3408.159 | 2114.419 | 1207.610 | 4193.947 | 4103.893 | 1709.171 |
| thinks things through before acting | 2293.467 | 3496.724 | 921.058 | 2567.920 | 6178.356 | 1173.872 |
| sees tasks through to the end | 2714.748 | 2750.505 | 1252.071 | 3913.745 | 4853.681 | 1159.918 |
| shares easily with others | 320.892 | 2157.657 | 4243.474 | 249.848 | 3307.127 | 6532.079 |
| considerate of others' feelings | 264.525 | 2394.977 | 4059.789 | 229.238 | 3116.490 | 6709.547 |
| helpful if someone is hurt, upset or ill | 248.686 | 2299.358 | 4183.479 | 171.179 | 1894.028 | 8002.533 |
| kind to younger children | 176.163 | 2065.279 | 4426.548 | 89.942 | 1526.035 | 8466.176 |
| often volunteers to help others | 692.605 | 2742.789 | 3293.886 | 227.943 | 3038.280 | 6732.471 |
| tends to play alone | 5400.803 | 942.850 | 384.088 | 7001.195 | 2386.578 | 635.668 |
| has at least one good friend | 5129.754 | 1206.458 | 385.372 | 8896.906 | 916.818 | 185.174 |
| generally liked by other children | 4844.935 | 1691.027 | 185.459 | 8561.428 | 1420.407 | 64.888 |
| picked on or bullied by other children | 6207.711 | 388.093 | 125.627 | 7606.693 | 1839.976 | 343.489 |
| gets on better with adults | 5238.889 | 1217.202 | 239.685 | 6394.006 | 2332.811 | 679.263 |

**Table S2: Descriptive statistics (weighted Ns by response category) for SDQ items at age 11**

| **Item** | **Teacher reports** | | | **Parent reports** | | |
| --- | --- | --- | --- | --- | --- | --- |
| **Paraphrase of item content** | **Not**  **true** | **Somewhat true** | **Certainly true** | **Not**  **true** | **Somewhat true** | **Certainly true** |
| complains of headaches, stomach aches, sickness | 5640.082 | 860.120 | 245.028 | 6712.918 | 2819.918 | 785.840 |
| often seems worried | 3983.505 | 2295.154 | 465.138 | 6202.424 | 3348.268 | 748.185 |
| often unhappy | 5347.401 | 1153.048 | 230.551 | 8393.912 | 1613.520 | 295.942 |
| nervous or clingy in new situations | 4908.987 | 1500.249 | 322.402 | 6174.560 | 3222.680 | 912.646 |
| many fears, easily scared | 5420.031 | 1125.459 | 170.138 | 7077.866 | 2595.978 | 587.779 |
| often has temper tantrums | 5797.188 | 677.961 | 268.668 | 5079.027 | 3578.592 | 1655.183 |
| generally obedient | 183.054 | 1263.373 | 5302.163 | 6144.261 | 3658.412 | 526.984 |
| fights with or bullies other children | 5848.908 | 735.525 | 162.163 | 9365.772 | 751.946 | 192.404 |
| steals from home, school or elsewhere | 6514.012 | 131.540 | 56.071 | 9922.486 | 248.406 | 103.352 |
| often lies or cheat | 5861.705 | 738.854 | 135.783 | 8170.776 | 1787.440 | 302.641 |
| restless, overactive, cannot stay still long | 5256.768 | 1068.956 | 421.520 | 6013.831 | 2876.039 | 1417.178 |
| constantly fidgeting | 5429.578 | 933.340 | 378.099 | 6940.944 | 2392.348 | 919.374 |
| easily distracted | 3806.413 | 2037.467 | 904.170 | 4449.870 | 4136.740 | 1713.248 |
| thinks things through before acting | 757.309 | 3166.157 | 2804.666 | 3064.573 | 5791.550 | 1379.012 |
| sees tasks through to the end | 878.320 | 2655.968 | 3206.887 | 3954.030 | 4916.511 | 1378.147 |
| shares easily with others | 245.234 | 1855.817 | 4643.266 | 321.351 | 2279.499 | 7740.917 |
| considerate of others' feelings | 272.577 | 2294.751 | 4176.979 | 367.355 | 2367.205 | 7547.865 |
| helpful if someone is hurt, upset or ill | 234.263 | 2159.862 | 4345.310 | 211.192 | 1679.472 | 8444.895 |
| kind to younger children | 110.090 | 1592.679 | 4995.034 | 96.830 | 1172.867 | 9074.387 |
| often volunteers to help others | 679.198 | 2642.453 | 3414.484 | 307.620 | 3253.578 | 6728.455 |
| tends to play alone | 5444.349 | 955.763 | 331.310 | 7103.255 | 2570.815 | 655.646 |
| has at least one good friend | 286.494 | 1083.737 | 5364.186 | 9022.401 | 999.802 | 295.697 |
| generally liked by other children | 200.939 | 1826.903 | 4724.912 | 8657.109 | 1526.266 | 132.738 |
| picked on or bullied by other children | 5832.948 | 754.519 | 158.785 | 6392.038 | 2835.397 | 828.476 |
| gets on better with adults | 5041.352 | 1418.308 | 248.337 | 7374.329 | 2261.545 | 538.701 |
